# Evaluation of WHO catalog of mutations and five WGS analysis tools for drug resistance prediction of *Mycobacterium tuberculosis* isolates from China

**DOI:** 10.1128/spectrum.03341-23

**Published:** 2024-06-21

**Authors:** Guiqing He, Qingyong Zheng, Jichan Shi, Lianpeng Wu, Bei Huang, Yang Yang

**Affiliations:** 1 Department of Infectious Diseases, Wenzhou Central Hospital, The Dingli Clinical College of Wenzhou Medical University, The Second Affiliated Hospital of Shanghai University, Wenzhou, Zhejiang, China; 2 Laboratory of Infectious Diseases, Wenzhou Central Hospital, The Dingli Clinical College of Wenzhou Medical University, The Second Affiliated Hospital of Shanghai University, Wenzhou, Zhejiang, China; 3 Department of Clinical Laboratory Medicine, Wenzhou Central Hospital, The Dingli Clinical College of Wenzhou Medical University, The Second Affiliated Hospital of Shanghai University, Wenzhou, Zhejiang, China; 4 Key Laboratory of Applied Technology on Green-Eco-Healthy Animal Husbandry of Zhejiang Province, Zhejiang Provincial Engineering Research Center for Animal Health Diagnostics and Advanced Technology, Zhejiang International Science and Technology Cooperation Base for Veterinary Medicine and Health Management, China-Australia Joint Laboratory for Animal Health Big Data Analytics, College of Animal Science and Technology and College of Veterinary Medicine of Zhejiang A&F University, Hangzhou, Zhejiang, China; Foundation for Innovative New Diagnostics, Geneva, Switzerland

**Keywords:** *Mycobacterium tuberculosis*, drug resistance, whole-genome sequencing, predictive capability

## Abstract

The continuous advancement of molecular diagnostic techniques, particularly whole-genome sequencing (WGS), has greatly facilitated the early diagnosis of drug-resistant tuberculosis patients. Nonetheless, the interpretation of results from various types of mutations in drug-resistant-associated genes has become the primary challenge in the field of molecular drug-resistance diagnostics. In this study, our primary objective is to evaluate the diagnosis accuracy of the World Health Organization (WHO) catalog of mutations and five WGS analysis tools (PhyResSE, Mykrobe, TB Profiler, Gen-TB, and SAM-TB) in drug resistance to 10 anti-*Mycobacterium tuberculosis* (MTB) drugs. We utilized the data of WGS collected between 2014 and 2017 in Zhejiang Province, consisting of 110 MTB isolates as detailed in our previous study. Based on phenotypic drug susceptibility testing (DST) results using the proportion method on Löwenstein-Jensen medium with antibiotics, we evaluated the predictive accuracy of genotypic DST obtained by these tools. The results revealed that the WHO catalog of mutations and five WGS analysis tools exhibit robust predictive capabilities concerning resistance to isoniazid, rifampicin, ethambutol, streptomycin, amikacin, kanamycin, and capreomycin. Notably, Mykrobe, SAM-TB, and TB Profiler demonstrate the most accurate predictions for resistance to pyrazinamide, prothionamide, and para-aminosalicylic acid, respectively. These findings are poised to significantly guide and influence future clinical treatment strategies and resistance monitoring protocols.

**IMPORTANCE:**

Whole-genome sequencing (WGS) has the potential for the early diagnosis of drug-resistant tuberculosis. However, the interpretation of mutations of drug-resistant-associated genes represents a significant challenge as the amount and complexity of WGS data. We evaluated the accuracy of the World Health Organization catalog of mutations and five WGS analysis tools in predicting drug resistance to first-line and second-line anti-TB drugs. Our results offer clinicians guidance on selecting appropriate WGS analysis tools for predicting resistance to specific anti-TB drugs.

## INTRODUCTION

Tuberculosis (TB), caused by the *Mycobacterium tuberculosis* (MTB), remains one of the major threats to global public health. According to the World Health Organization (WHO) Global Tuberculosis Report 2022, approximately 10.6 million people fell ill with TB, and 1.6 million people died of TB in 2021 (1). Despite multiple strategies that have been taken to prevent the TB epidemic, the global treatment success rate for drug-resistant TB (DR-TB) remains low at 60%, making it a key hurdle in achieving TB elimination (1).

Early diagnosis and treatment of DR-TB are crucial for preventing death and reducing disease transmission. However, conventional drug susceptibility testing (DST), while accurate, has limitations such as complexity, time consumption, and technical demands, which restrict its widespread use (2). In recent years, rapid molecular assays using molecular biological techniques have emerged as promising alternatives. The Xpert MTB/RIF or Xpert Ultra is a PCR diagnostic test that detects *rpoB* gene mutation in MTB related to rifampicin (RFP) resistance, offering higher sensitivity and shorter diagnostic time compared to DST (3). Nevertheless, this method can only identify a limited number of resistance-associated gene mutations, and its results may not be comprehensive or entirely reliable. More recently, genotypic resistance prediction from MTB sequences using whole-genome sequencing (WGS) has been in rapid development. WGS not only enables faster determination of drug resistance compared to conventional DST and molecular assays but also accurately predicts resistance to various anti-TB drugs without requiring specialized infrastructure (4). While sequencing technology has matured, data analysis remains a challenge for predicting drug resistance using WGS technology. Fortunately, several tools have been developed to aid in the accurate and rapid identification and prediction of DR-TB, including PhyResSE (5), Mykrobe (6), TB Profiler (7), Gen-TB (8), and SAM-TB (9). These tools vary in their scope and specificity, from generic to more refined applications. Besides detecting drug-resistance mutations and identifying MTB lineages, they can be also used for species identification of nontuberculous mycobacteria (NTM) or combined these two functions (9). Moreover, the WHO has published a catalog of MTB complex mutations associated with drug resistance, serving as a global standard for interpreting molecular information on resistance predictions (10). The performance of the WHO mutations catalog or WGS online tools to predict drug resistance depends on the integrity and accuracy of the drug-resistant mutation database employed. However, it is worth noting that variations in DR-TB mutations may exist across different countries, regions, ethnic groups, and populations. Further studies are needed to validate the predictive value of WGS analysis tools for different populations and regions.

In this study, our main objective was to evaluate the accuracy of the WHO mutations catalog and five WGS analysis tools (PhyResSE, Mykrobe, TB Profiler, Gen-TB, and SAM-TB) in predicting resistance to both first-line and second-line drugs. To achieve this, we utilized DST and WGS data from a total of 110 MTB isolates, which had been previously published in our earlier study (11). The aim was to compare the predictions made by these tools against the known resistance profiles obtained through DST and WGS analysis of the isolates. This assessment was critical in determining the reliability and effectiveness of the WHO catalog and the WGS tools in accurately predicting drug resistance in MTB, thereby contributing to the advancement of TB treatment and management strategies.

## RESULTS

### Prediction of resistance to first-line drugs

**Fig 1:**
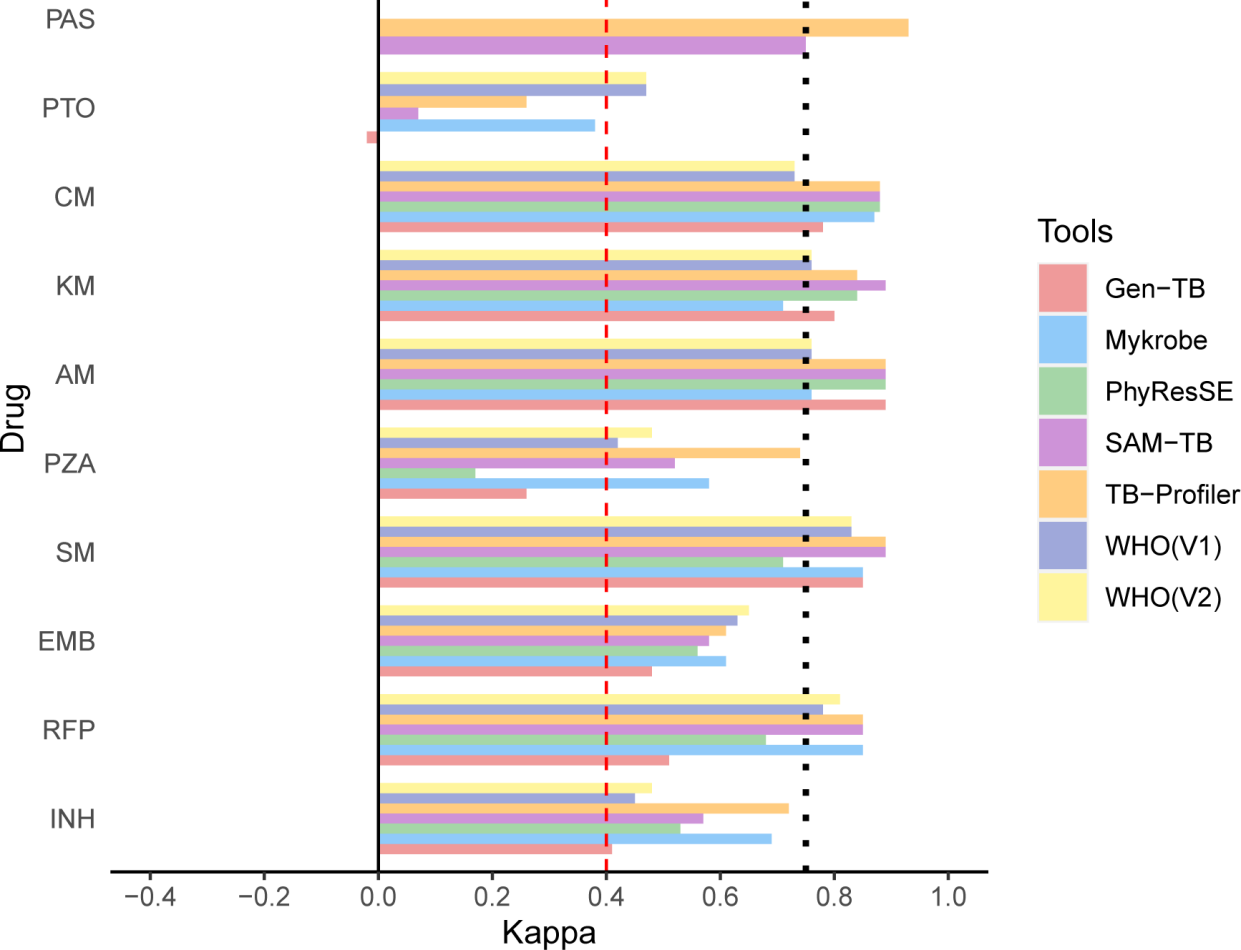
Forest plot diagram of Kappa value for the drug-resistant predictive capability of WHO catalogs of mutations and five WGS analysis tools (*n* = 110). All antibiotics, except PZA and PTO, were tested using the proportion method on a commercial Löwenstein-Jensen medium with antibiotics. The susceptibility of MTB to PZA and PTO was evaluated using an automated Mycobacterial Growth Indicator Tube 960system. WGS data were analyzed using the WHO catalog of mutations [first version (V1) issued in 2021 and second version (V2) issued in 2023] and five user-friendly online tools (TB Profiler v3.0.7, SAM-TB, GenTB v7.24.0, PhyResSE v1.0, and Mykrobe v0.12.1.). PAS, para-aminosalicylic acid; AM, amikacin; KM, kanamycin; CM, capreomycin; PTO, prothionamide.

**Fig 2:**
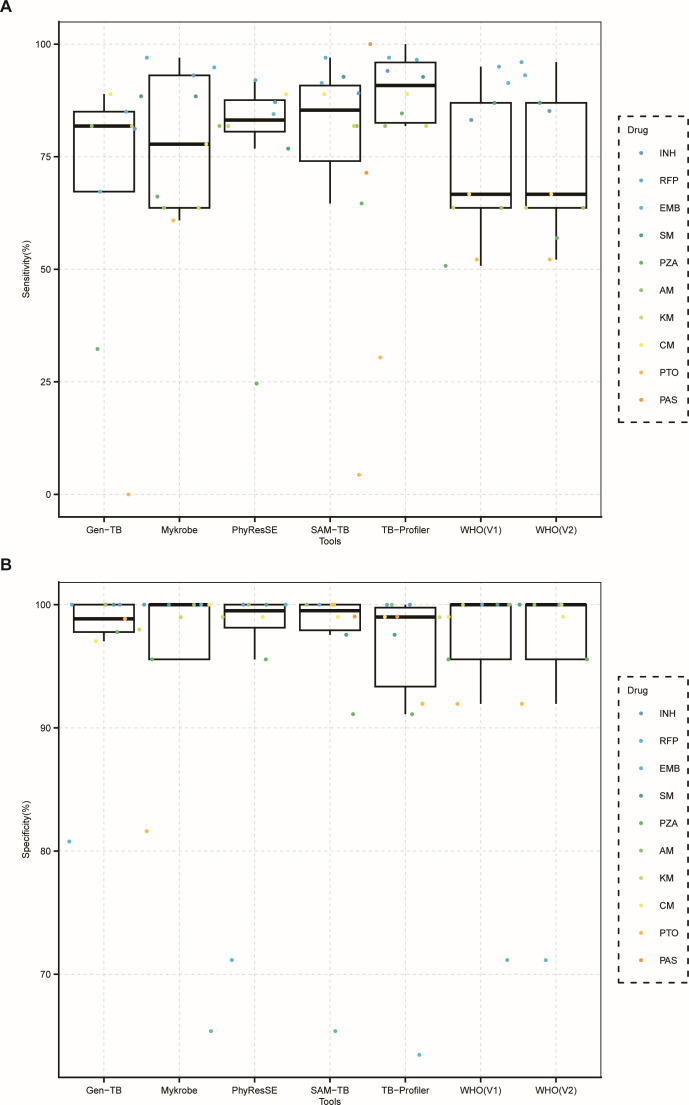
Diagnostic accuracy of WHO catalogs of mutations and five WGS analysis tools for drugs (*n* = 110). Box plot displaying sensitivity (**A**) and specificity (**B**) to predict drug resistance for WHO catalogs of mutations [first version (V1) issued in 2021 and second version issued (V2) in 2023] and five user-friendly online tools (TB Profiler v3.0.7, SAM-TB, GenTB v7.24.0, PhyResSE v1.0, and Mykrobe v0.12.1.). PAS, para-aminosalicylic acid; AM, amikacin; KM, kanamycin; CM, capreomycin; PTO, prothionamide.

We assessed the predictive performance of the WHO catalog of mutations (first and second versions) and five WGS online analysis tools by phenotypic culture-based DST results (Table 1; Fig. 1 and 2). WHO catalog of mutations and five online analysis tools all showed high performance in predicting the specificity of isoniazid (INH) and RFP resistance, with all achieving 100% accuracy. However, there were some differences in sensitivity. Among them, TB Profiler exhibited the highest sensitivity, while Gen-TB had the lowest sensitivity. Gen-TB showed the best specificity in predicting ethambutol (EMB) resistance, while TB Profiler exhibited the best sensitivity in predicting EMB resistance. All tools achieved a specificity of over 97% in predicting streptomycin (SM) resistance and a sensitivity of over 86% (except for PhyResSE, which had a sensitivity of 76.81%). TB Profiler had the best sensitivity in predicting pyrazinamide (PZA) resistance, while the sensitivity predicted by other tools was 66% or lower. All tools predicted a specificity of over 91% for PZA resistance. Overall, the accuracy of tools in PZA resistance prediction varied significantly, with TB Profiler demonstrating the most accurate to others.

**TABLE 1:** Performance of the WHO catalogs of mutations and five WGS analysis tools for predicting drug resistance of MTB (*n* = 110)^*a*^

Drug	Tools	Phenotypically resistant		Phenotypically sensitive		Sensitivity (95% CI)	Specificity (95% CI)	PPV (95% CI)	NPV (95% CI)	Consistency	Kappa
		Genetically resistant	Genetically sensitive	Genetically resistant	Genetically sensitive						
INH	TB Profiler	95	6	0	9	94.06% (87.01–97.56)	100% (62.88–100)	100% (95.16–100)	60% (32.89–82.54)	94.55%	0.72
	SAM-TB	90	11	0	9	89.11% (80.96–94.17)	100% (62.88–100)	100% (94.90–100)	45% (23.83–67.95)	90%	0.57
	Gen-TB	82	19	0	9	81.19% (71.93–88.02)	100% (62.88–100)	100% (94.42–100)	32.14% (16.58–52.43)	82.73%	0.41
	PhyResSE	88	13	0	9	87.13% (78.64–92.70)	100% (62.88–100)	100% (94.79–100)	40.91% (21.48–63.32)	88.18%	0.53
	Mykrobe	94	7	0	9	93.07% (85.76–96.93)	100% (62.88–100)	100% (95.11–100)	56.25% (30.55–79.25)	93.64%	0.69
	WHO (V1)	84	17	0	9	83.17% (74.13–89.61)	100% (62.88–100)	100% (94.55–100)	34.62% (17.94–55.64)	84.55%	0.45
	WHO (V2)	86	15	0	9	85.15% (76.37–91.18)	100% (62.88–100)	100% (94.67–100)	37.50% (19.55–59.24)	86.36%	0.48
RFP	TB Profiler	97	3	0	10	97% (90.85–99.22)	100% (65.55–100)	100% (95.25–100)	76.92% (45.98–93.84)	97.27%	0.85
	SAM-TB	97	3	0	10	97% (90.85–99.22)	100% (65.55–100)	100.00 (95.25–100)	76.92% (45.98–93.84)	97.27%	0.85
	Gen-TB	85	15	0	10	85% (76.15–91.09)	100% (65.55–100)	100% (94.61–100)	40% (21.81–61.11)	86.36%	0.51
	PhyResSE	92	8	0	10	92% (84.39–96.23)	100% (65.55–100)	100% (95.01–100)	55.56% (31.35–77.60)	92.73%	0.68
	Mykrobe	97	3	0	10	97% (90.85–99.22)	100% (65.55–100)	100% (95.25–100)	76.92 (45.98–93.84)	97.27%	0.85
	WHO (V1)	95	5	0	10	95% (88.17–98.14)	100% (65.55–100)	100% (95.16–100)	66.67% (38.69–87.01)	95.45%	0.78
	WHO-2 (V2)	96	4	0	10	96% (89.49–98.71)	100% (65.55–100)	100% (95.21–100)	71.43% (42.00–90.42)	96.36%	0.81
EMB	TB Profiler	56	2	19	33	96.55% (87.05–99.41)	63.46% (48.91–76.03)	74.67% (63.08–83.69)	94.29% (79.48–99.00)	80.91%	0.61
	SAM-TB	53	5	18	34	91.38% (80.28–96.78)	65.38% (50.39–77.67)	74.65% (62.69–83.90)	87.18% (71.77–95.18)	79.09%	0.58
	Gen-TB	39	19	10	42	67.24% (53.54–78.65)	80.77% (67.03–89.92)	79.59% (65.24–89.28)	68.85% (55.56–79.76)	73.64%	0.48
	PhyResSE	49	9	15	37	84.48% (72.07–92.23)	71.15% (56.73–82.45)	76.56% (64.02–85.88)	80.43% (65.62–90.14)	78.18%	0.56
	Mykrobe	55	3	18	34	94.83% (84.70–98.65)	65.38% (50.84–77.67)	75.34% (63.62–84.36)	91.89% (76.98–97.88)	80.91%	0.61
	WHO (V1)	54	4	15	37	93.10% (82.45–97.77)	71.15% (56.73–82.45)	78.26% (66.39–86.94)	90.24% (75.94–96.83)	82.73%	0.65
	WHO (V2)	54	4	15	37	93.10% (82.45–97.77)	71.15% (56.73–82.45)	78.26% (66.39–86.94)	90.24% (75.94–96.83)	82.73%	0.65
SM	TB Profiler	64	5	1	40	92.75% (83.21–97.30)	97.56% (85.59–99.87)	98.46% (90.60–99.92)	88.89% (75.15–95.84)	94.55%	0.89
	SAM-TB	64	5	1	40	92.75% (83.21–97.30)	97.56% (85.59–99.87)	98.46% (90.60–99.92)	88.89% (75.15–95.84)	94.55%	0.89
	Gen-TB	61	8	0	41	88.41% (77.89–94.51)	100% (89.33–100)	100% (92.62–100)	83.67% (69.80–92.20)	92.73%	0.85
	PhyResSE	53	16	0	41	76.81% (64.82–85.78)	100% (89.33–100)	100% (91.58–100)	71.93% (58.25–82.64)	85.45%	0.71
	Mykrobe	61	8	0	41	88.41% (77.89–94.51)	100% (89.33–100)	100% (92.62–100)	83.67% (69.80–92.20)	92.73%	0.85
	WHO (V1)	60	9	0	41	86.96% (76.18–93.50)	100% (89.33–100)	100% (92.50–100)	82% (68.08–90.95)	91.82%	0.83
	WHO (V2)	60	9	0	41	86.96% (76.18–93.50)	100% (89.33–100)	100% (92.50–100)	82% (68.08–90.95)	91.82%	0.83
PZA	TB Profiler	55	10	4	41	84.62% (73.06–91.99)	91.11% (77.87–97.11)	93.22% (82.73–97.81)	80.39% (66.45–89.71)	87.27%	0.74
	SAM-TB	42	23	4	41	64.62% (51.70–75.80)	91.11% (77.87–97.11)	91.30% (78.31–97.18)	64.06% (51.03–75.40)	75.45%	0.52
	Gen-TB	21	44	1	44	32.31% (21.54–45.18)	97.78% (86.77–99.88)	95.45% (75.12–99.76)	50% (39.23–60.77)	59.09%	0.26
	PhyResSE	16	49	2	43	24.62% (15.13–37.13)	95.56% (83.63–99.22)	88.89% (63.93–98.05)	46.74% (36.37–57.39)	53.64%	0.17
	Mykrobe	43	22	2	43	66.15% (53.26–77.13)	95.56% (83.64–99.22)	95.56% (83.64–99.23)	66.15% (53.26–77.13)	78.18%	0.58
	WHO (V1)	33	32	2	43	50.77% (38.19–63.26)	95.56% (83.64–99.23)	94.29% (79.48–99.00)	57.33% (45.40–68.51)	69.09%	0.42
	WHO (V2)	37	28	2	43	56.92% (44.08–68.94)	95.56% (83.64–99.23)	94.87% (81.37–99.11)	60.56% (48.24–71.74)	72.73%	0.48
AM	TB Profiler	9	2	0	99	81.82% (47.76–96.79)	100% (95.35–100)	100% (62.88–100)	98.02% (92.34–99.66)	98.18%	0.89
	SAM-TB	9	2	0	99	81.82% (47.76–96.79)	100% (95.35–100)	100% (62.88–100)	98.02% (92.34–99.66)	98.18%	0.89
	Gen-TB	9	2	0	99	81.82% (47.76–96.79)	100% (95.35–100)	100% (62.88–100)	98.02% (92.34–99.66)	98.18%	0.89
	PhyResSE	9	2	0	99	81.82% (47.76–96.79)	100% (95.35–100.00)	100% (62.88–100)	98.02% (92.34–99.66)	98.18%	0.89
	Mykrobe	7	4	0	99	63.64% (31.61–87.63)	100% (95.35–100)	100% (56.09–100)	96.12% (89.78–98.75)	96.36%	0.76
	WHO (V1)	7	4	0	99	63.64% (31.61–87.63)	100% (95.35–100)	100% (56.09–100)	96.12% (89.78–98.75)	96.36%	0.76
	WHO (V2)	7	4	0	99	63.64% (31.61–87.63)	100% (95.35–100)	100% (56.09–100)	96.12% (89.78–98.75)	96.36%	0.76
KM	TB Profiler	9	2	1	98	81.82% (47.76–96.79)	98.99% (93.70–99.95)	90% (54.12–99.48)	98% (92.26–99.65)	97.27%	0.84
	SAM-TB	9	2	0	99	81.82% (47.76–96.79)	100% (95.35–100)	100% (62.88–100)	98.02% (92.34–99.66)	98.18%	0.89
	Gen-TB	9	2	2	97	81.82% (47.76–96.79)	97.98% (92.19–99.65)	81.82% (47.76–96.79)	97.98% (92.19–99.65)	96.36%	0.80
	PhyResSE	9	2	1	98	81.82% (47.76–96.79)	98.99% (93.70–99.95)	90% (54.12–99.48)	98% (92.26–99.65)	97.27%	0.84
	Mykrobe	7	4	1	98	63.64% (31.61–87.63)	98.99% (93.70–99.95)	87.50% (46.68–99.34)	96.08% (89.69–98.74)	95.45%	0.71
	WHO (V1)	7	4	0	99	63.64% (31.61–87.63)	100% (95.35–100)	100% (56.09–100)	96.12% (89.78–98.75)	96.36%	0.76
	WHO (V2)	7	4	0	99	63.64% (31.61–87.63)	100% (95.35–100)	100% (56.09–100)	96.12% (89.78–98.75)	96.36%	0.76
CM	TB Profiler	8	1	1	100	88.89% (50.67–99.42)	99.01% (93.82–99.95)	88.89% (50.67–99.42)	99.01% (93.82–99.95)	98.18%	0.88
	SAM-TB	8	1	1	100	88.89% (50.67–99.42)	99.01% (93.82–99.95)	88.89% (50.67–99.42)	99.01% (93.82–99.95)	98.18%	0.88
	Gen-TB	8	1	3	98	88.89% (50.67–99.42)	97.03% (90.94–99.23)	72.73% (39.32–92.67)	98.99% (93.70.99.95)	96.36%	0.78
	PhyResSE	8	1	1	100	88.89% (50.67–99.42)	99.01% (93.82–99.95)	88.89% (50.67–99.42)	99.01% (93.82–99.95)	98.18%	0.88
	Mykrobe	7	2	0	101	77.78% (40.19–96.06)	100% (95.43–100)	100% (56.09–100)	98.06% (92.48–99.66)	98.18%	0.87
	WHO (V1)	6	3	1	100	66.67% (30.92–90.96)	99.01% (93.82–99.95)	85.71% (42.01–99.25)	97.09% (91.10–99.24)	96.36%	0.73
	WHO (V2)	6	3	1	100	66.67% (30.92–90.96)	99.01% (93.82–99.95)	85.71% (42.01–99.25)	97.09% (91.10–99.24)	96.36%	0.73
PTO	TB Profiler	7	16	7	80	30.43% (14.06–53.01)	91.95% (83.60–96.43)	50% (24.04–75.96)	83.33% (74.04–89.89)	79.09%	0.26
	SAM-TB	1	22	0	87	4.35% (0.22–23.97)	100% (94.73–100)	100% (5.46–100)	79.82% (70.82–86.66)	80%	0.07
	Gen-TB	0	23	1	86	0% (0.00–17.81)	98.85% (92.87–99.94)	0% (0.00–94.54)	78.90% (69.82–85.89)	78.18%	(0.02)
	PhyResSE	/	/	/	/	/	/	/	/	/	/
	Mykrobe	14	9	16	71	60.87% (38.78–79.53)	81.61% (71.55–88.81)	46.67% (28.80–65.36)	88.75% (79.24–94.41)	77.27%	0.38
	WHO (V1)	12	11	7	80	52.17% (31.08–72.58)	91.95% (83.60–96.43)	63.16% (38.63–82.77)	87.91% (78.99–93.52)	83.64%	0.47
	WHO (V2)	12	11	7	80	52.17% (31.08–72.58)	91.95% (83.60–96.43)	63.16% (38.63–82.77)	87.91% (78.99–93.52)	83.64%	0.47
PAS	TB Profiler	7	0	1	102	100% (56.09–100)	99.03 (93.93–99.95)	87.50% (46.68–99.34)	100% (95.48–100)	99.09%	0.93
	SAM-TB	5	2	1	102	71.43% (30.26–94.89)	99.03% (93.93–99.95)	83.33% (36.48–99.12)	98.08% (92.55–99.67)	97.27%	0.75
	Gen-TB	/	/	/	/	/	/	/	/	/	/
	PhyResSE	/	/	/	/	/	/	/	/	/	/
	Mykrobe	/	/	/	/	/	/	/	/	/	/
	WHO (V1)	/	/	/	/	/	/	/	/	/	/
	WHO (V2)	/	/	/	/	/	/	/	/	/	/

^*a*^ All antibiotics, except PZA and PTO, were tested using the proportion method on a commercial Löwenstein-Jensen medium with antibiotics. The susceptibility of MTB to PZA and PTO was evaluated using an automated Mycobacterial Growth Indicator Tube 960system. WGS data were analyzed using the WHO catalog of mutations [first version (V1) issued in 2021 and second version (V2) issued in 2023] and five user-friendly online tools (TB Profiler v3.0.7, SAM-TB, GenTB v7.24.0, PhyResSE v1.0, and Mykrobe v0.12.1.). The numbers in parentheses represent 95% confidence intervals. PAS, para-aminosalicylic acid; AM, amikacin; KM, kanamycin; CM, capreomycin; PTO, prothionamide; PPV, positive predictive value; NPV, negative predictive value; CI, confidence interval; /, not applicable.

### Prediction of resistance to second-line drugs

All tools predict a specificity between 98% and 100% for the second-line injectable drugs amikacin (AM) and kanamycin (KM; Table 1; Fig. 2). The sensitivity for resistance to these drugs is 63.64% for Mykrobe and both versions of the WHO catalog of mutations and 81.82% for the other four tools. For capreomycin (CM) resistance, all tools predict a specificity above 97.03% with a sensitivity of 88.89%, except for Mykrobe (77.78%) and both versions of the WHO catalog of mutations (66.67%). SAM-TB performs the best in predicting resistance to prothionamide (PTO), with a specificity of 100%, followed by Gen-TB with a specificity of 98.85%. There are significant differences among the tools in predicting sensitivity to PTO resistance, with Mykrobe achieving the highest sensitivity at 60.87%. Only TB Profiler and SAM-TB can predict resistance to para-aminosalicylic acid (PAS) with a specificity of 99.03%, but there are significant differences in sensitivity, with values of 100% (TB Profiler) and 71.43% (SAM-TB).

## DISCUSSION

The early diagnosis of drug-resistant tuberculosis based on molecular diagnostic techniques is of crucial importance for the effective control of TB. In recent years, due to the rapid advancement of sequencing technologies, WGS has gained prominence as a vital method for drug-resistance testing in tuberculosis (12, 13). Its key advantage lies in its ability to swiftly detect all known drug-resistant mutations. In 2018, the WHO recommended the use of WGS for the rapid diagnosis of drug-resistant tuberculosis (14). However, the main bottleneck of the current application of WGS lies in the analysis and interpretation of the massive data generated, as well as the lack of standardized, comprehensive coverage of gene mutations and their associations with drug resistance (15). Given this, numerous analysis tools have been developed to analyze WGS data and predict drug resistance. In 2021, the WHO also published the catalog of drug-resistant mutations to promote the mutual recognition of genotypic DST, phenotypic DST, and sequencing data, as well as to enhance the understanding of mutations associated with drug-resistant phenotypes. Moreover, WHO has recently published an update to the mutations catalog in 2023. In this study, we performed a comparison of the WHO catalog of mutations (version 1.0 and 2.0) and five user-friendly WGS analysis tools for the effectiveness of predicting drug resistance in MTB. The comparison involved 10 types of anti-tuberculosis drugs, including all first-line drugs (INH, RFP, EMB, SM, and PZA) and some second-line drugs (AM, KM, CM, PTO, and PAS). Overall, WGS tools demonstrate better prediction performance for first-line drugs than second-line drugs.

Compared to the WHO catalog of mutations and other WGS analysis tools, Gen-TB has poor performance in predicting the sensitivity of INH and RFP resistance. The catalog of INH-resistant mutations Gen-TB used only includes *katG*_S315N, *katG*_S315T, and *fabG1*_C-15T. In addition, resistant mutations of *inhA* gene (*inhA*_G154A, *inhA*_T770C, and *inhA*_C777T) are listed into the WHO catalog of mutations, which might explain why the WHO catalog of mutations achieves better sensitivity (83.17%) than Gen-TB (81.19%). Nearly all (95% or more) RFP-resistant strains harbor mutations within the 81 bp RFP-resistance-determining region in *rpoB* gene (16). The reason for the poor sensitivity of Gen-TB for RFB resistance is the lack of the mutations S431G, Q432L, D435Y, H445D, H445Q, and L452P in *ropB* gene which have been reported in previous studies (17–19). The different resistant mutations that were collected into the catalogs used for the interpretation of variants made the difference performance of WGS analysis tools. Specifically, we found that the predictive accuracy of WGS analysis tools for EMB, SM, and PZA showed large differences, for which the genetic basis of resistance is more complex than for RFP and INH. For example, PZA resistance is not only primarily associated with a mutation in *pncA* and its promoter region but also associated with a mutation in *rpsA*, *panD*, *clpC1*, and other unidentified mechanism of genes (20).

The WHO catalog of mutations and five WGS analysis tools achieve high accuracy in predicting resistance to AM, KM, and CM (consistency of more over 85%), but the results are not ideal for PTO. Only TB Profiler and SAM-TB could predict resistance for PAS with a good concordance rate of the phenotypic DST and WGS. PhyResSE cannot predict resistance for PTO, while the WHO catalog of mutations and other four tools perform good specificity but poor sensitivity for PTO resistance.

WHO has recently published an update to the mutations catalog (second version) in 2023 (21). We compared the first and second versions to investigate whether the updated catalog has improved the predictive ability for drug resistance. Following the update of the WHO catalog, we observed improvements in the prediction accuracy for first-line drugs INH, RFP, and PZA. This was evidenced by the inclusion of resistance mutation sites such as *inhA*_G779T for INH, *rpoB*_431S_432QinsR for RFP, and *pncA*_C14W for PZA. The mutation inhA_G779T for INH, previously not considered a resistance mutation in the first version of the WHO catalog, is now recognized as such in the second version, possibly due to updates in WGS data analysis methodology. The second version of the WHO catalog continues to retain variations with an allele frequency of ≥75% (which was 90% in the first version) for further association analysis, and it also provides additional assessment for variations with an allele frequency threshold reduced to 25% (22). Conversely, there were minimal alterations in the prediction results for second-line drugs.

Fluoroquinolones (FQs) are recognized for their critical role, especially in combination therapies with emerging drugs such as bedaquiline (BDQ) and linezolid (LZD) (23). The exclusion of FQs was a deliberate decision based on the retrospective nature of our data collection, which limited our access to certain drug classes. However, we acknowledge that this exclusion may introduce a limitation in our study’s ability to fully elucidate the drug-resistance profiles predicted by WGS. The impact of not accounting for FQs could potentially affect the generalizability of our findings, particularly in settings where FQs are more commonly prescribed. Future research endeavors should aim to incorporate a broader spectrum of antimicrobial agents, including FQs, and refine WGS prediction models to better account for the intricate resistance profiles of Mtb strains (24). Additionally, our research also lacks the predictive capability of WGS for new anti-TB drugs (such as LZD and BDQ) resistance. This is partly because the acquisition time of our strains predates the launch of these new drugs in China, and no resistant strains were identified in the preliminary phenotypic results. Moreover, the mechanisms of resistance to these new drugs are not well understood, and there is poor consistency between existing genotype-based resistance analyses and phenotypic resistance (25). Therefore, this study did not discuss research on genotype-based prediction of resistance to these new drugs.

The present study provides a better understanding of the performance of the WHO catalog of mutations and five WGS analysis tools in predicting drug resistance in MTB. WHO catalog of mutations and five WGS analysis tools exhibit robust predictive capabilities concerning resistance to INH, RFP, EMB, SM, AM, KM, and CM. Mykrobe, SAM-TB, and TB Profiler demonstrate the most accurate predictions for resistance to PZA, PTO, and PAS, respectively. These findings will serve as critical points of reference and guidance for future clinical treatment and resistance monitoring.

## MATERIALS AND METHODS

### Evaluation data set

Our data set consisted of 100 multi-drug resistant (MDR) strains and 10 non-MDR strains. These strains were randomly selected from a pool of over 6,000 clinical samples, among which 329 strains were identified as MDR. All strains were obtained from Wenzhou Sixth People’s Hospital, Wenzhou Central Hospital Medical Group, Zhejiang Province, China, between 1 January 2014 and 30 June 2017. Both the phenotypic DST results and WGS data were available for these strains. This set of strains has been described in our previous study (11). Initially, a colloidal gold assay (Genesis Biodetection and Biocontrol Ltd., Hangzhou, Zhejiang Province, China) was routinely employed for the detection of the MPB64 antigen, allowing differentiation between MTB and NTM. All NTM isolates were systematically excluded from the study. Following this initial screening, DNA extraction was performed, and the presence of MTB was further validated through PCR amplification and Sanger sequencing of the 16s rRNA. Importantly, in cases where a patient presented with multiple isolates, caution was exercised by excluding the earlier isolates from the analysis.

### Phenotypic DST and critical concentration

The DST of all clinical isolates and the reference strain MTB H37Rv to 13 anti-TB drugs were carried out according to the Clinical and Laboratory Standards Institute (CLSI) and WHO guidelines. All antibiotics, except PZA and PTO, were tested using the proportion method on a commercial Löwenstein-Jensen medium with antibiotics (Baso, Zhuhai, Guangzhou Province, China). The critical concentrations were 0.2 mg/L for INH, 40.0 mg/L for RFP, 2.0 mg/L for EMB, 4.0 mg/L for SM, 30.0 mg/L for AM, 30.0 mg/L for KM, 40.0 mg/L for CM, and 1.0 mg/L for PAS, respectively, according to the CLSI guidelines and WHO guidelines (26, 27). The results were determined after 3 weeks of incubation at 37°C. The susceptibility of MTB to PZA and PTO was evaluated using an automated Mycobacterial Growth Indicator Tube 960system (Becton Dickinson Diagnostic Systems, Franklin Lakes, NJ, USA) according to the manufacturer’s instructions at critical concentrations of 100.0 and 2.5 mg/L, respectively. The limitation of technical feasibility and reproducibility of the phenotypic DST of PZA, EMB, and PTO required the DST to be performed at least twice for these three drugs. If the two results were inconsistent, a third test was performed. All experiments using live MTB were performed in a biosafety level 2 plus laboratory. Among these strains, 100 of them were MDR strains, while the remaining strains were non-MDR strains.

### Data analysis

WGS data of 110 strains were analyzed using five user-friendly online tools: TB Profiler v3.0.7 (https://tbdr.lshtm.ac.uk/), SAM-TB (http://samtb.szmbzx.com), GenTB v7.24.0 (https://gentb.hms.harvard.edu), PhyResSE v1.0 (http://phyresse.org), and Mykrobe v0.12.1 (https://github.com/mykrobe-tools/mykrobe). Additionally, we utilized the “Catalog of mutations in MTB complex and their association with drug resistance,” issued by WHO in 2021 (first version) (28) and 2023 (second version) to analyze our WGS data. All resistance-related mutations including borderline resistance mutations have been listed in Tables S1 and S2.

### Statistical analyses

The phenotypic DST was used as the gold standard to calculate sensitivity, specificity, positive predictive value, and negative predictive value, and their 95% confidence intervals as well as consistency and kappa value using VassarStats (http://vassarstats.net/index.html).

## AFTER EPUB

[This article was published on 21 June 2024 with an error in the Discussion. The error was corrected in the current version, posted on 11 July 2024.]

## SUPPLEMENTAL MATERIAL

Table S1 spectrum.03341-23-s0001.xlsx Overview of the resistance-related mutations.

Table S2 spectrum.03341-23-s0002.xlsx Frequency of resistance associated mutations and genotypic results.

ASM does not own the copyrights to Supplemental Material that may be linked to, or accessed through, an article. The authors have granted ASM a non-exclusive, world-wide license to publish the Supplemental Material files. Please contact the corresponding author directly for reuse.

## References

[1] Bagcchi S. 2023. WHO’s global tuberculosis report 2022. Lancet Microbe 4:e20. doi:10.1016/S2666-5247(22)00359-7 36521512

[2] Singh BK, Sharma SK, Sharma R, Sreenivas V, Myneedu VP, Kohli M, Bhasin D, Sarin S. 2017. Diagnostic utility of a line probe assay for multidrug resistant-TB in smear-negative pulmonary tuberculosis. PLoS One 12:e0182988. doi:10.1371/journal.pone.0182988 28829779 PMC5568731

[3] Denkinger CM, Schumacher SG, Boehme CC, Dendukuri N, Pai M, Steingart KR. 2014. Xpert MTB/RIF assay for the diagnosis of extrapulmonary tuberculosis: a systematic review and meta-analysis. Eur Respir J 44:435–446. doi:10.1183/09031936.00007814 24696113

[4] Shea J, Halse TA, Lapierre P, Shudt M, Kohlerschmidt D, Van Roey P, Limberger R, Taylor J, Escuyer V, Musser KA. 2017. Comprehensive whole-genome sequencing and reporting of drug resistance profiles on clinical cases of Mycobacterium tuberculosis in New York state. J Clin Microbiol 55:1871–1882. doi:10.1128/JCM.00298-17 28381603 PMC5442544

[5] Feuerriegel S, Schleusener V, Beckert P, Kohl TA, Miotto P, Cirillo DM, Cabibbe AM, Niemann S, Fellenberg K. 2015. PhyResSE: a web tool delineating Mycobacterium tuberculosis antibiotic resistance and lineage from whole-genome sequencing data. J Clin Microbiol 53:1908–1914. doi:10.1128/JCM.00025-15 25854485 PMC4432036

[6] Hunt M, Bradley P, Lapierre SG, Heys S, Thomsit M, Hall MB, Malone KM, Wintringer P, Walker TM, Cirillo DM, et al.. 2019. Antibiotic resistance prediction for Mycobacterium tuberculosis from genome sequence data with Mykrobe. Wellcome Open Res 4:191. doi:10.12688/wellcomeopenres.15603.1 32055708 PMC7004237

[7] Phelan JE, O’Sullivan DM, Machado D, Ramos J, Oppong YEA, Campino S, O’Grady J, McNerney R, Hibberd ML, Viveiros M, Huggett JF, Clark TG. 2019. Integrating informatics tools and portable sequencing technology for rapid detection of resistance to anti-tuberculous drugs. Genome Med 11:41. doi:10.1186/s13073-019-0650-x 31234910 PMC6591855

[8] Gröschel MI, Owens M, Freschi L, Vargas R Jr, Marin MG, Phelan J, Iqbal Z, Dixit A, Farhat MR. 2021. GenTB: a user-friendly genome-based predictor for tuberculosis resistance powered by machine learning. Genome Med 13:138. doi:10.1186/s13073-021-00953-4 34461978 PMC8407037

[9] Yang T, Gan M, Liu Q, Liang W, Tang Q, Luo G, Zuo T, Guo Y, Hong C, Li Q, Tan W, Gao Q. 2022. SAM-TB: a whole genome sequencing data analysis website for detection of Mycobacterium tuberculosis drug resistance and transmission. Brief Bioinform 23:bbac030. doi:10.1093/bib/bbac030 35211720 PMC8921607

[10] Walker TM, Miotto P, Köser CU, Fowler PW, Knaggs J, Iqbal Z, Hunt M, Chindelevitch L, Farhat M, Cirillo DM, et al.. 2022. The 2021 WHO catalogue of Mycobacterium tuberculosis complex mutations associated with drug resistance: a genotypic analysis. Lancet Microbe 3:e265–e273. doi:10.1016/S2666-5247(21)00301-3 35373160 PMC7612554

[11] Chen X, He G, Wang S, Lin S, Chen J, Zhang W. 2019. Evaluation of whole-genome sequence method to diagnose resistance of 13 anti-tuberculosis drugs and characterize resistance genes in clinical multi-drug resistance Mycobacterium tuberculosis isolates from China. Front Microbiol 10:1741. doi:10.3389/fmicb.2019.01741 31417530 PMC6685394

[12] Witney AA, Gould KA, Arnold A, Coleman D, Delgado R, Dhillon J, Pond MJ, Pope CF, Planche TD, Stoker NG, Cosgrove CA, Butcher PD, Harrison TS, Hinds J. 2015. Clinical application of whole-genome sequencing to inform treatment for multidrug-resistant tuberculosis cases. J Clin Microbiol 53:1473–1483. doi:10.1128/JCM.02993-14 25673793 PMC4400773

[13] Holicka Y, Tagliani E, Cirillo DM, Nikolayevskyy V. 2022. Utility of the whole genome sequencing based methodologies in routine European tuberculosis reference laboratory network setting. Tuberculosis (Edinb) 134:102185. doi:10.1016/j.tube.2022.102185 35247779

[14] World Health Organization. 2018. The use of next-generation sequencing technologies for the detection of mutations associated with drug resistance in Mycobacterium tuberculosis complex: technical guide. World Health Organization, Geneva. https://apps.who.int/iris/handle/10665/274443.

[15] Kwong JC, McCallum N, Sintchenko V, Howden BP. 2015. Whole genome sequencing in clinical and public health microbiology. Pathology 47:199–210. doi:10.1097/PAT.0000000000000235 25730631 PMC4389090

[16] Shea J, Halse TA, Kohlerschmidt D, Lapierre P, Modestil HA, Kearns CH, Dworkin FF, Rakeman JL, Escuyer V, Musser KA. 2021. Low-level rifampin resistance and rpoB mutations in Mycobacterium tuberculosis: an analysis of whole-genome sequencing and drug susceptibility test data in New York. J Clin Microbiol 59:e01885-20. doi:10.1128/JCM.01885-20 32999007 PMC8579749

[17] Donnabella V, Martiniuk F, Kinney D, Bacerdo M, Bonk S, Hanna B, Rom WN. 1994. Isolation of the gene for the beta subunit of RNA polymerase from rifampicin-resistant Mycobacterium tuberculosis and identification of new mutations. Am J Respir Cell Mol Biol 11:639–643. doi:10.1165/ajrcmb.11.6.7946393 7946393

[18] Ramaswamy SV, Dou SJ, Rendon A, Yang Z, Cave MD, Graviss EA. 2004. Genotypic analysis of multidrug-resistant Mycobacterium tuberculosis isolates from Monterrey, Mexico. J Med Microbiol 53:107–113. doi:10.1099/jmm.0.05343-0 14729930

[19] Sajduda A, Brzostek A, Poplawska M, Augustynowicz-Kopec E, Zwolska Z, Niemann S, Dziadek J, Hillemann D. 2004. Molecular characterization of rifampin- and isoniazid-resistant Mycobacterium tuberculosis strains isolated in Poland. J Clin Microbiol 42:2425–2431. doi:10.1128/JCM.42.6.2425-2431.2004 15184414 PMC427864

[20] Rajendran A, Palaniyandi K. 2022. Mutations associated with pyrazinamide resistance in Mycobacterium tuberculosis: a review and update. Curr Microbiol 79:348. doi:10.1007/s00284-022-03032-y 36209317

[21] World Health Organization. 2023. Catalogue of mutations in Mycobacterium tuberculosis complex and their association with drug resistance. 2nd ed. World Health Organization, Geneva. https://www.who.int/publications/i/item/9789240082410.

[22] World Health Organization. 2018. The use of next-generation sequencing technologies for the detection of mutations associated with drug resistance in Mycobacterium tuberculosis complex: technical guide. World Health Organization, Geneva. https://www.who.int/publications/i/item/WHO-CDS-TB-2018.19.

[23] Fan C, Eedara BB, Sinha S, Uddin MKM, Doyle C, Banu S, Das SC. 2024. Triple combination dry powder formulation of pretomanid, moxifloxacin, and pyrazinamide for treatment of multidrug-resistant tuberculosis. Int J Pharm 654:123984. doi:10.1016/j.ijpharm.2024.123984 38461874

[24] Parmanik A, Das S, Kar B, Bose A, Dwivedi GR, Pandey MM. 2022. Current treatment strategies against multidrug-resistant bacteria: a review. Curr Microbiol 79:388. doi:10.1007/s00284-022-03061-7 36329256 PMC9633024

[25] Barilar I, Battaglia S, Borroni E, Brandao AP, Brankin A, Cabibbe AM, Carter J, Chetty D, Cirillo DM, Claxton P, et al.. 2024. Quantitative measurement of antibiotic resistance in Mycobacterium tuberculosis reveals genetic determinants of resistance and susceptibility in a target gene approach. Nat Commun 15:488. doi:10.1038/s41467-023-44325-5 38216576 PMC10786857

[26] World Health Organization. 2018. Technical report on critical concentrations for drug susceptibility testing of medicines used in the treatment of drug-resistant tuberculosis. WHO/CDS/TB/2018.5. World Health Organization, Geneva. https://www.who.int/publications/i/item/WHO-CDS-TB-2018.5.

[27] CLSI. 2011. Susceptibility testing of mycobacteria, nocardiae, and other aerobic actinomycetes. 2nd ed. CLSI Document M24-A2. Clinical and Laboratory Standards Institute, Wayne, PA. 31339680

[28] World Health Organization. 2021. Catalogue of mutations in Mycobacterium tuberculosis complex and their association with drug resistance. World Health Organization, Geneva. https://www.who.int/publications/i/item/9789240028173.

